# Characterization of Polyurethane Shape Memory Polymer and Determination of Shape Fixity and Shape Recovery in Subsequent Thermomechanical Cycles

**DOI:** 10.3390/polym14214775

**Published:** 2022-11-07

**Authors:** Maria Staszczak, Mana Nabavian Kalat, Karol Marek Golasiński, Leszek Urbański, Kohei Takeda, Ryosuke Matsui, Elżbieta Alicja Pieczyska

**Affiliations:** 1 Institute of Fundamental Technological Research, Polish Academy of Sciences, 02-106 Warsaw, Poland; 2 Aichi Institute of Technology, Toyota City 470-0392, Japan

**Keywords:** polyurethane shape memory polymer, thermomechanical loading program, shape fixity, shape recovery

## Abstract

Multifunctional polyurethane shape memory polymers (PU-SMPs) have been of increasing interest in various applications. Here we report structure characterization, detailed methodology, and obtained results on the identification of functional properties of a thermoset PU-SMP (MP4510) with glass transition temperature of 45 °C. The stable, chemically crosslinked network of this thermoset PU-SMP results in excellent shape memory behavior. Moreover, the proximity of the activation temperature range of this smart polymer to room and body temperature enables the PU-SMP to be used in more critical industrial applications, namely fast-response actuators. The thermomechanical behavior of a shape memory polymer determines the engineering applications of the material. Therefore, investigation of the shape memory behavior of this class of commercial PU-SMP is of particular importance. The conducted structural characterization confirms its shape memory properties. The shape fixity and shape recovery properties were determined by a modified experimental approach, considering the polymer’s sensitivity to external conditions, i.e., the temperature and humidity variations. Three thermomechanical cycles were considered and the methodology used is described in detail. The obtained shape fixity ratio of the PU-SMP was approximately 98% and did not change significantly in the subsequent cycles of the thermomechanical loading due to the stability of chemical crosslinks in the thermoset materials structure. The shape recovery was found to be approximately 90% in the first cycle and reached a value higher than 99% in the third cycle. The results confirm the effect of the thermomechanical training on the improvement of the PU-SMP shape recovery after the first thermomechanical cycle as well as the effect of thermoset material stability on the repeatability of the shape memory parameters quantities.

## 1. Introduction

Shape memory polymers (SMPs) are stimuli-responsive materials that have attracted great attention as smart materials due to their high shape recovery characteristics—high elastic deformation, easy manipulation, light weight, and low density—in comparison with the considerably heavier and more expensive shape memory alloys [1,2]. The SMP is capable of restoring the original shape from the deformed temporary shape, which has been changed by mechanical loadings and temperature changes. The restoration process in general happens by exposing the SMP to an external stimulus, such as heat [3,4,5,6], light [7,8], magnetic field [9], solvents [10], moisture [6,11], laser, microwave heating [8,12], or other factors.

Heat is the most frequent external stimulus used for recovery activation of the original shape of the SMP. The glass transition temperature (*T_g_*) of a thermo-responsive SMP is determined as the activation temperature. It plays an important role in determining the functional behavior of the SMP, due to the different molecular structures of the polymer segments above and below the *T_g_* [13,14]. The high elastic modulus below *T_g_*, which is the result of frozen micro-Brownian motion, decreases dramatically at the temperature above *T_g_*, which allows the internal stresses to be released, leading to the process of shape recovery [15,16].

Among the thermo-responsive SMPs, thermoset and thermoplastic polyurethane shape memory polymers (PU-SMPs) are especially distinguished, owing to their good shape memory and high mechanical properties, i.e., relatively high strength and fatigue properties [1,2,3]. Thermoset and thermoplastic PU-SMPs are copolymers consisting of hard and soft segments arranged inhomogeneously and randomly in the polymer chains, leading to the formation of a two-phase structure. The interactions between the hard and soft segments below and above *T_g_* are responsible for the fixation of the temporarily deformed shape as well as the recovery of the original shape [17,18,19,20,21]. The hard segments act as net points, consisting of physical or/and chemical crosslinks stabilizing the original shape, whereas the soft segments are in charge of molecular switches and reversible phases [13,22]. Thermoplastic PU-SMPs possess linear molecular structure, which is physically crosslinked by hydrogenic, van der Waals, and polar bonds. Although, thermoplastic PU-SMPs demonstrate more deformability and are easy to process and recycle, their unstable network leads to low stress strength and mechanical damages [2,14], while the chemically crosslinked network of thermoset PU-SMPs by covalent bonds induces high mechanical strength as well as thermal, solvent, and dimensional permanence [23,24,25,26].

These unique properties, demonstrated at various temperatures, allow the PU-SMP to be used for various new functional applications [14,27,28] in various fields, including aviation and aerospace industries [14,29,30,31,32], robotics, sensors, and actuators [33,34,35], biomedical devices such as self-expandable vascular stents [36], smart textiles used in intelligent sport clothes and underwear, smart pillows, mattresses, and sport shoe insoles [37,38].

The ability of the PU-SMP to fix its deformed shape below *T_g_* and recover its original shape above *T_g_* defines the shape memory behavior of the material, which can be determined by shape fixity and shape recovery properties. The shape fixity and shape recovery parameters of PU-SMP were firstly proposed by H. Tobushi and S. Hayashi, followed by other researchers working on shape memory polymers. The parameters have been determined by using the proposed formulas and the experimental data obtained in the process of the so-called program of thermomechanical loading [14,15,39].

The influence of the number of thermomechanical cycles on the polymer shape memory properties has been studied in order to investigate the stability of shape memory parameters in the multiple shape memory cycles and predict the PU-SMP behavior in the potential applications. Tobushi et al. found the shape fixity and shape recovery of the casting film of a commercial PU-SMP with *T_g_* of 55 °C independent of number of cycles, but not in the initial thermomechanical cycles [15]. They noticed that shape recovery reached approximately 98% after the fourth cycle, indicating the importance of the mechanical training effect on shape memory parameters. They obtained the same results for the PU-SMP foam with *T_g_* of 55 °C [16]. In fact, mechanical training helps the specimen reach a stable functional behavior after the initial cycles. Ohki et al. observed that by increasing the number of cycles in the PU-SMP/glass–fiber composite, the shape recovery rate remained unchanged after the second cycle [40]. Pringpromsuk et al. noticed that both shape fixity and shape recovery of pure and plasticized thermoplastic PU-SMP with *T_g_* of 65 °C were improved by increasing the number of cycles up to three cycles, attributed to the training effect [41]. Santiago et al. observed the opposite result in the commercial PU-SMP Tecoflex, such that the shape recovery ratio was decreased with subsequent thermomechanical cycles [42]. The decrease of shape recovery ratio with increasing the number of cycles was also obtained by Li et al. in the thermoplastic PU-SMP with *T_g_* of 45 °C provided from the same producer as reported in this paper [43].

The abovementioned literature provided data regarding the influence of repeating the thermomechanical cycle on shape memory parameters of the thermoplastic PU-SMPs. Thermoset PU-SMP can demonstrate better and more repeatable shape memory parameters due to the chemically crosslinked 3D structure. Due to the increasing use of smart polymers in different application fields, the thermomechanical and structural properties of unique commercial PU-SMPs require investigation to be employed in applications.

The proposed work concerns comprehensive experimental research and provides technical information about the thermo-sensitive thermoset polyurethane shape memory polymer PU-SMP (MP4510) with *T_g_* = 45 °C, produced by the SMP Technologies Inc., Tokyo, Japan, including the investigation of its structure and the functional properties. Moreover, the effect of temperature on the mechanical behavior of the PU-SMP during loading–unloading in the thermal chamber is investigated, while the temperatures varied from below *T_g_* to above *T_g_*. The temperature range in which this class of PU-SMP is activated and demonstrates shape memory behavior is close to room and body temperature, enabling the PU-SMP to be used in new design of smart orthopedics and post-traumatic rehabilitation devices, where good fit and compliance are expected, but also high strength is a very important parameter. Similar requirements must be met in the case of critical industrial applications, namely fast-response actuators. In order to examine the stability of the thermoset SMPs properties, the repeatability of shape memory parameters of this thermoset PU-SMP in three thermomechanical cycles is investigated. A slightly modified experimental procedure is proposed to determine shape fixity and shape recovery in the thermomechanical loading program. To this end, additional elements of the setup for increasing measurement accuracy are introduced. Reproducible conditions were created by controlling the specimen temperature at various points inside the thermal chamber and keeping a constant level of humidity in each test. The shape fixity and shape recovery parameters were measured with high accuracy and investigated in one and in three subsequent cycles of the thermomechanical loading.

## 2. Materials and Methods

### 2.1. Materials and Specimens

**Figure 1:**
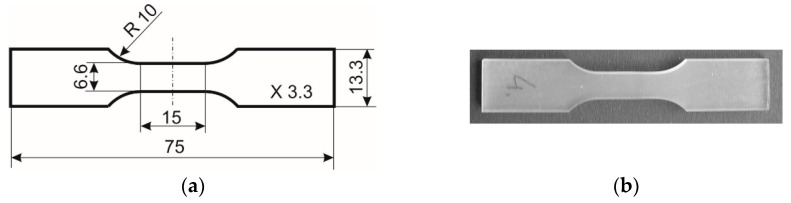
(**a**) Technical drawing and (**b**) picture of polyurethane shape memory polymer PU-SMP specimen.

The investigation was conducted on the thermoset polyurethane shape memory polymer PU-SMP (MP4510) with *T_g_* = 45 °C, produced by the SMP Technologies Inc., Tokyo, Japan. The specimens were cut from the PU-SMP sheet with a thickness of 3.2 mm. The technical drawing and picture of the specimen used during the thermomechanical loading program are presented in Figure 1a,b, respectively.

### 2.2. Structural Characterization Methods

In order to better understand the behavior of the material in various conditions and to select the appropriate parameters for the thermomechanical loading program, the research began with an extensive program of the PU-SMP structural characterization. To this end, dynamic mechanical analysis (DMA), differential scanning calorimetry (DSC), scanning electron microscopy (SEM), atomic force microscopy (AFM), and ultrasound testing (UT) were conducted.

The **DMA** was performed in the bending mode with a frequency of force oscillation of 1 Hz and a heating rate of 2 °C/min by the PerkinElmer Diamond instrument (Waltham, MA, USA) equipped with a dual cantilever clamp. The bar shape specimen with a length of 20 mm, width of 12 mm, and thickness of 3.5 mm was used.

The **DSC** was carried out using a PerkinElmer Pyris 1 DSC calorimeter (Waltham, MA, USA). PU-SMP samples of about 10 mg were heated from −10 °C to +210 °C at the rate of 10 °C/min.

The **SEM** investigation of the PU-SMP surfaces was conducted at room temperature using a scanning electron microscope JEOL JSM-6480 (JEOL Ltd., Tokyo, Japan). The results were analyzed with a JEOL program. The sample surface had been sprayed with a thin layer of carbon before the observation in order to obtain better conductivity.

The **AFM** was performed in the tapping mode with a scan frequency of 0.25 Hz on the Hysitron TI 950 TriboIndenter (Minneapolis, MN, USA) with AFM Q-Scope 250 head (Quesant) to investigate the microstructure and phase separation by detecting the sample topography.

The **UT** was conducted in order to determine the value of Young’s modulus and Poisson’s ratio with high accuracy by a non-destructive method. Pulse–echo technique was used. Generation and detection of ultrasonic pulses were performed with a Panametrics-NDT™ EPOCH 4 (Waltham, MA, USA) ultrasonic flaw detector. Conducting the measurement was possible due to the significantly high glass transition temperature of the PU-SMP *T_g_* = 45 °C, exhibiting its relatively high rigidity at room temperature.

### 2.3. Thermomechanical Characterization Methods

#### 2.3.1. Mechanical Behavior Characterization at Various Temperatures

In order to study the influence of temperature on the mechanical behavior of the PU-SMP and to choose the appropriate experimental parameters for the thermomechanical loading program, the PU-SMP was subjected to the tension conducted at the constant strain rate of 10^−2^ s^−1^ in isothermal conditions at various temperatures. To this end, the specimens were loaded up to the strain value of 0.117 in a thermal chamber below *T_g_* (25 °C, 30 °C, and 35 °C), at approximately *T_g_* (45 °C), and above *T_g_* (65 °C), attributed to glassy, glass transition, and rubbery regions of the polymer, respectively. Then, the specimens were unloaded with the same strain rate to, approximately, the zero-force level.

#### 2.3.2. Thermomechanical Loading Program-Modified Setup and Experimental Procedure

**Figure 2:**
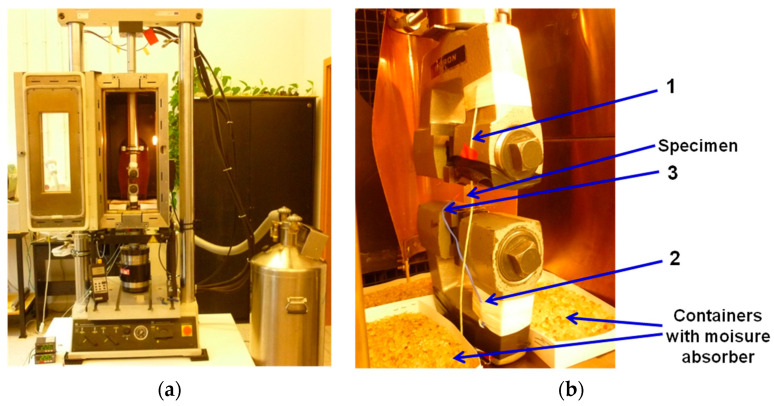
Photographs of (**a**) experimental setup: a testing machine with a thermal chamber; and (**b**) PU-SMP specimen in grips of testing machine with additional thermocouples (1, 2, 3) and moisture absorbers in the boxes.

The program of PU-SMP thermomechanical loading contained mechanical and thermal loading cycles conducted in a thermal chamber coupled to the testing machine. In the experiment, a MTS 858 testing machine (MTS Systems Corporation, Eden Prairie, MN, USA) was used, equipped with an Instron SFL 3119-406 thermal chamber (Figure 2a). As designed by the producer, the temperature in the thermal chamber was measured by using only one thermocouple located near the air inlet. According to the data given in the chamber manual, the system is automatically controlled and can be used in a temperature range from −100 °C to +350 °C; the temperature setting accuracy is ±5 °C.

Shape memory polymers are extremely sensitive to external factors, especially temperature and humidity variations [2]. Therefore, in this study, in order to uniform the temperature of the specimens and increase the accuracy of the obtained results, three additional thermocouples were placed in the thermal chamber: the first on the upper grip of the testing machine (1), the second on the lower grip (2), and the third in the vicinity of the specimen gauge length (3) (Figure 2b). After setting the required temperature in the chamber, the temperature of each thermocouple was controlled and the specimen-loading was begun only when the temperature differences between the three thermocouples were recorded to be lower than 1 °C. The accuracy of the specimen temperature measurement was ±1 °C.

Introducing additional systems to control the temperature in the vicinity of the specimen allows all the experimental programs for all PU-SMP specimens to be carried out under much more uniform conditions.

Furthermore, as reported by Yang et al., exposing the PU-SMP to the air at room temperature or immersing the specimen in water results in a significant decrease in Young’s modulus, *T_g_*, and shape fixity [11]. Because the absorbed humidity in the air penetrates the PU-SMP matrix, acts as a plasticizer, and increases the mobility of the polymer chain, in ether-based PU-SMP, absorbed water may weaken the hydrogen bonds, resulting in the decrease of *T_g_* [44]. The moisture significantly affects the behavior of the PU-SMP; therefore, in this study moisture absorbers were placed in the boxes on the bottom plate of the testing machine (Figure 2b) to minimalize the influence of the humidity. In particular, silica gel with a chemical composition of SiO_2_ ≈ 97%; Al_2_O_3_ ≈ 2.9%, and a moisture indicator of 0.1% was used as the moisture absorber in order to reduce rapid humidity changes under radical temperature variations and to uniform and maintain a similar level of humidity in the thermal chamber in each experiment.

**Figure 3:**
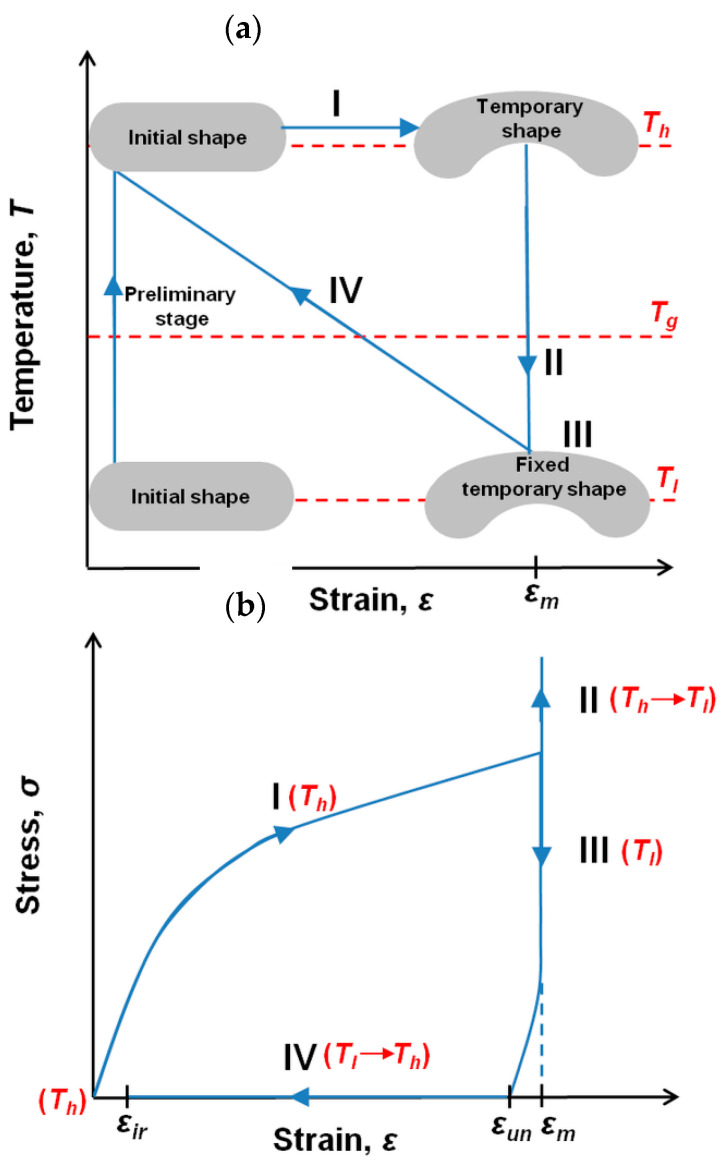
Schematic diagrams showing subsequent stages I–IV of the PU-SMP thermomechanical loading program: (**a**) temperature and (**b**) stress vs. strain curves.

The PU-SMP specimen with a gauge length of 15 mm, shown in Figure 1, was subjected to a thermomechanical loading program using the modified experimental setup introduced above and presented in Figure 2. The schematic of the program showing the preliminary and the subsequent stages of the thermomechanical loading (I–IV) is described in Table 1 and depicted in Figure 3.

At the preliminary stage, the specimen was heated up to the high temperature of *T_h_* = 65 °C (*T_g_* + 20 °C) at a heating rate of 4 °C/min. After that, the specimen was loaded by tension with a strain rate of $\dot{\varepsilon }$ = 10^−3^ s^−1^ at the temperature *T_h_* until obtaining the maximum strain of 20% (*ε_m_*) (stage I). Then, while maintaining the maximum strain value (*ε_m_*), the specimen was cooled down to the low temperature *T_l_* = 25 °C (*T_g_* − 20 °C) at the cooling rate of 7 °C/min in order to fix its temporary shape (stage II). Afterward, the specimen was unloaded to the zero-force value at *T_l_* (stage III) at the strain rate of $\dot{\varepsilon }$ = 10^−3^ s^−1^. The strain value obtained after unloading was close to the maximum strain value *ε_m_*, showing the shape fixity property of the PU-SMP at temperatures below *T_g_*. Then, the specimen was heated again from *T_l_* to *T_h_* at the heating rate of 4 °C/min under no-load conditions, leading to the recovery of the PU-SMP original shape (stage IV). The PU-SMP specimen almost recovered its shape; however, a residual strain *ε_ir_* was recorded. 

## 3. Results and Discussion

### 3.1. PU-SMP Structural Characterization Results and Analysis

#### 3.1.1. Dynamic Mechanical Analysis

**Figure 4:**
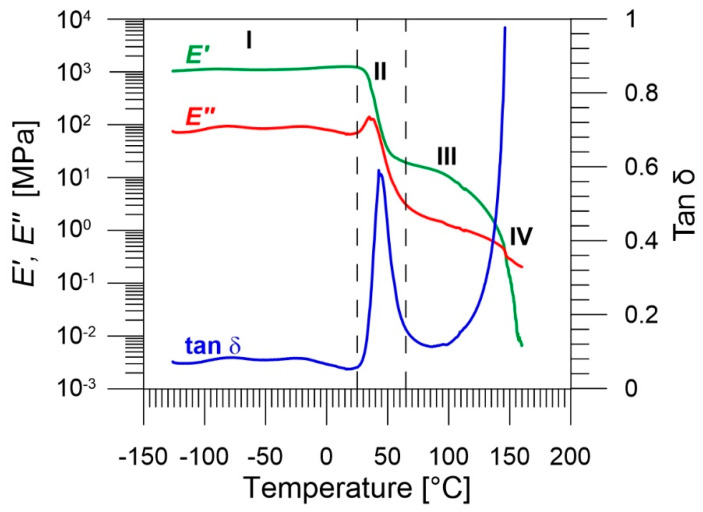
DMA results for PU-SMP: storage modulus *E*′, loss modulus *E*″, and loss factor tan *δ* vs. temperature: I—glassy, II—glass transition, III—rubbery, and IV—flowing regions.

The DMA diagram of the PU-SMP (Figure 4) presents the temperature dependence of storage modulus *E*′, loss modulus *E*″, and loss factor tan *δ*, which allowed us to distinguish (I) glassy, (II) glass transition, (III) rubbery, and (IV) flowing regions. The obtained results provided in Table 2 represent the parameters, leading to the shape memory behavior of the PU-SMP [45]. The high value of storage modulus in the glassy region *E*′*_g_* = 1250 MPa is due to structure of the thermoset PU-SMP and ensures a high shape fixity of the polymer during the cooling down to the temperature below *T_g_* and the subsequent unloading at this temperature. The low value of the storage modulus in the rubbery region *E*′*_r_* = 12.1 MPa, provides a large deformation in the rubbery state and a high elastic recovery at high temperatures. Moreover, the high ratio of *E*′*_g_*/*E*′*_r_* = 103 enables an easy deformation at temperatures higher than *T_g_* and high resistance to deformation at temperatures lower than *T_g_*. For a sharp transition from the glassy to the rubbery state, a difference of at least two orders of magnitude in the elastic modulus values below and above *T_g_* is needed. This makes the material sensitive enough to temperature variations. The glass transition temperature, which is determined as the peak of loss tangent (tan *δ*), is approximately equal to 45 °C and confirms the value provided by the producer.

#### 3.1.2. Differential Scanning Calorimetry

**Figure 5:**
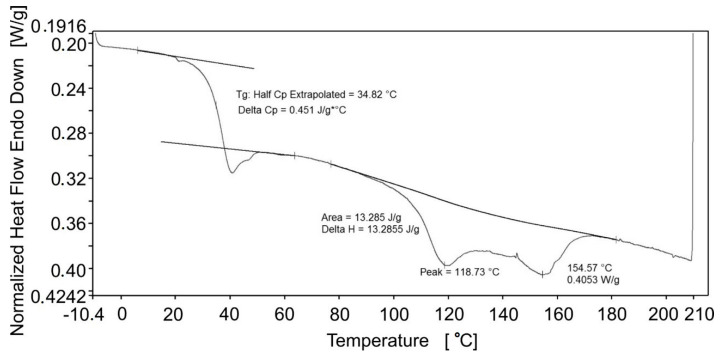
The average heat flow vs. temperature obtained during first heating from three DSC tests.

The average heat flow vs. temperature curve obtained during first heating from three DSC tests is shown in Figure 5. A step change in the heat flow at 34.82 °C indicates the glass transition temperature range of the polymer. The *T_g_* of the polymer derived from DSC is approximately 35 °C, which differs from DMA results and the value provided by the producer (45 °C). However, the differences in results are not contradictory. Different heating rates in DMA and DSC can affect the determined value of the glass transition temperature. Moreover, when determined by different methods, there are usually discrepancies between the values of the glass transition temperature, which result from the different methodology of these tests and measurements. The DMA is a mechanical test, while the DSC is based on measuring the change in heat capacity of a static sample. Furthermore, the glass transition temperature is a temperature range and its value depends on the method of determination. Afterward, during further heating, two additional endothermic peaks appear at temperatures 119 °C and 155 °C, which are likely associated with degradation of some meso-phase. The initial XRD studies showed a lack of any diffraction peaks.

#### 3.1.3. Scanning Electron Microscopy

**Figure 6:**
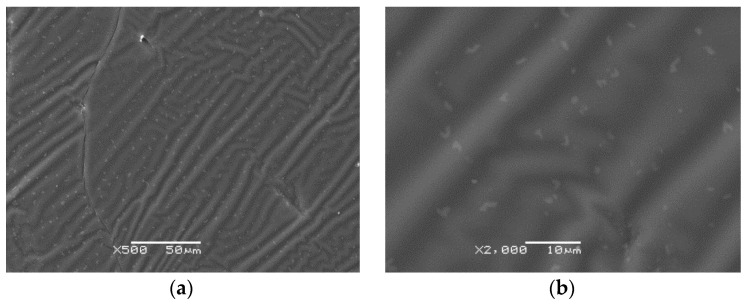
SEM images of PU-SMP sample at magnification of: (**a**) ×500 and (**b**) ×2000.

The SEM investigation was performed for three PU-SMP samples. The photos showed a similar surface for all samples. The SEM images obtained for one of the samples at magnifications of ×500 and ×2000 are shown in Figure 6a and Figure 6b, respectively.

The effect of block copolymer morphology of the PU-SMP bulk is visible on its surface in Figure 6. As shown in the images, microphase separation for the investigated PU-SMP sample is also observed. At a magnification of ×500, the dispersion of some irregularities (lighter areas) in a continuous phase is presented (Figure 6a). It can be supposed that a continuous phase is built with the amorphous part of the soft and intermediate phases. The hard phase and soft phase form some domains with irregular shapes as lighter spots which are more visible at a magnification of ×2000, as shown in Figure 6b.

The segregated structure of shape memory polyurethane composed of hard and soft segments was also confirmed in the literature by structural analysis [45,46]. A separation in two phases occurred in the PU-SMP due to the poor compatibility between the hard segments and soft segments [47,48]. Hard domains act as physical and/or chemical crosslinks and as a reinforcing filler in a soft-segment-rich soft matrix.

#### 3.1.4. Atomic Force Microscopy

**Figure 7:**
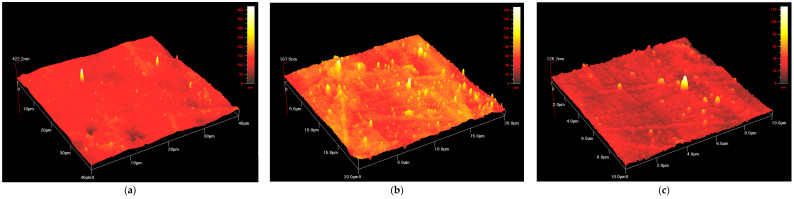
AFM images obtained for PU-SMP at scan range: (**a**) 40 μm × 40 μm; (**b**) 20 μm × 20 μm; and (**c**) 10 μm × 10 μm.

The sample surfaces of three PU-SMP samples were scanned in three areas using AFM. The photos were similar for all samples. The topographic images of one of scanned PU-SMP sample surface are presented as follows: 40 μm × 40 μm (Figure 7a), 20 μm × 20 μm (Figure 7b), and 10 μm × 10 μm (Figure 7c). The results demonstrate that the surface topography of the PU-SMP is significantly influenced by its morphology. Figure 7 reveals the phase separation in PU-SMP represented by darker and brighter zones. The darker zones can be attributed to the continuous phase of soft domains, while the brighter zones can represent the hard domains, which are embedded heterogeneously in the soft domain matrix [49].

#### 3.1.5. Ultrasound Testing

The determination of the elastic modulus by UT is based on the fact that ultrasonic waves are elastic waves. Therefore, the velocities of the waves directly depend on the elastic properties and the propagation medium density. Assuming that the investigated polymer is isotropic, it was sufficient to measure the velocities of ultrasonic waves in two perpendicular directions of the specimen cross-section. Then, the following equations were used for the determination of the PU-SMP Young’s modulus *E* and Poisson’s ratio *ν*, respectively:

(1) $$E=\rho \frac{\left(3V_{L}^{2}V_{T}^{2}-4V_{T}^{4}\right)}{\left(V_{L}^{2}-V_{T}^{2}\right)},$$

(2) $$\nu =\frac{\left(\frac{1}{2}V_{L}^{2}-V_{T}^{2}\right)}{\left(V_{L}^{2}-V_{T}^{2}\right)}.$$

where *ρ* is the mass density, *V_L_* is the velocity of longitudinal ultrasonic waves, and *V_T_* is the velocity of transversal ultrasonic waves [50].

Four plate-shaped samples with dimensions of 42 mm × 30 mm × 3.4 mm were investigated. The obtained UT results of PU-SMP are shown in Table 3. The determined mean value of Young’s modulus at room temperature by ultrasound testing is equal to 3177 MPa, while the average value of Poisson’s ratio is equal to 0.412. These elastic properties are key parameters in engineering design and materials development which help to understand the mechanical behavior of materials. A precise measurement for both constants is necessary to describe elastic deformation in some applications, such as the design of mechanically loaded components [51,52,53]. 

### 3.2. PU-SMP Thermomechanical Characterization—Results and Analysis

#### 3.2.1. Demonstration of PU-SMP Shape Memory Properties

**Figure 8:**
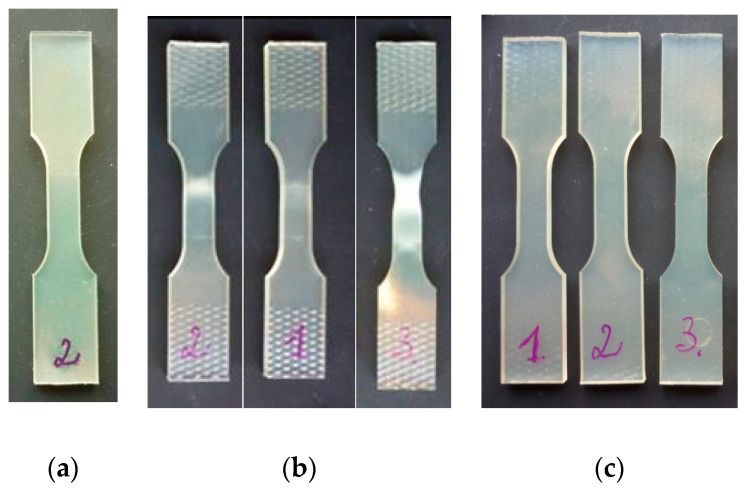
Demonstration of shape memory properties of PU-SMP: (**a**) specimen at the initial state before loading; (**b**) specimens after loading under various deformation programs; and (**c**) after heating at 65 °C.

Demonstration of the shape memory properties of PU-SMP specimens monitored by the authors is presented in Figure 8a–c. The photograph of a specimen taken at the initial state is shown in Figure 8a, while three specimens, marked by 1, 2, and 3, after various programs of loading and deformation conducted at room temperature, are presented in Figure 8b. The significant effects of deformation, depending on the loading program, are noticed in each specimen. Subsequently, all specimens were heated under no load at the temperature of 65 °C (*T_g_* + 20 °C) for 30 min to recover their original shape. The same specimens (1, 2, and 3) after the thermal recovery are shown in Figure 8c.

It is quite visible that the macroscopic effects of deformation vanished by exposing the deformed specimens to the heating at the temperatures above *T_g_*; all PU-SMP specimens almost recover their initial shapes and dimensions, due to the shape memory properties.

#### 3.2.2. Influence of Temperature on PU-SMP Mechanical Behavior 

**Figure 9:**
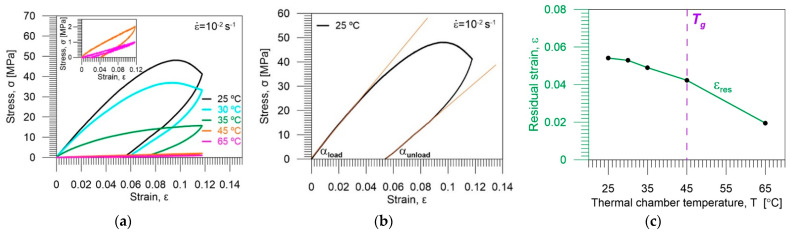
(**a**) Stress *σ* vs. strain *ε* obtained during tension loading–unloading of PU-SMP with glass transition temperature *T_g_* = 45 °C at strain rate of 10^−2^ s^−1^ at various temperatures (*T_g_* − 20 °C; *T_g_* − 15 °C; *T_g_* − 10 °C; and *T_g_*; *T_g_* + 20 °C); insert shows curves obtained at *T_g_* and *T_g_* + 20 °C with a larger scale; (**b**) stress *σ* vs. strain obtained at *T_g_* − 20 °C, showing the method of determination of loading–unloading tangent modulus; and (**c**) residual strain *ε_res_* vs. temperature.

The PU-SMP mechanical behavior was investigated during the tension process conducted at a constant strain rate of 10^−2^ s^−1^ in isothermal conditions at various temperatures below *T_g_* (25 °C, 30 °C, and 35 °C), at approximately *T_g_* (45 °C) and above *T_g_* (65 °C). The obtained results, namely the mean stress vs. strain curves elaborated from three tests for each temperature, are presented in Figure 9a, while Figure 9b separately shows data obtained at 25 °C. Moreover, the residual strain *ε_res_* vs. temperature curve obtained after unloading of PU-SMP is demonstrated in Figure 9c.

As can be noticed in Figure 9a, the PU-SMP mechanical behavior at temperatures below *T_g_* (25 °C, 30 °C, and 35 °C) is entirely different from the polymer behavior at *T_g_* and at temperature above *T_g_* (65 °C). By comparing Young’s modulus of the PU-SMP in different temperatures (slope of the initial linear part of the stress-strain curve in Figure 9a), it has been evidently confirmed that the elastic modulus of the polymer is much higher when the specimen is deformed at temperatures below *T*_g_. At low temperatures, the polymer is in the glassy state and behaves similarly to elasto-plastic material [2,14,31]. As can be seen in Figure 9b, the initial linear part of the loading curve is almost parallel to the terminal linear part of the unloading curve, while it becomes less parallel with increasing temperatures at which the mechanical behavior is investigated (Table 4). At temperatures below *T_g_*, the soft segments, which act as reversible phase, do not possess enough energy to obtain good mobility; therefore, the lower the loading temperature, the higher the stress values the polymer needs to become deformed.

Above *T_g_*, the PU-SMP reaches the rubbery state and becomes deformed easily. Thus, the stress values and elastic moduli obtained at *T_g_* and *T_g_* + 20 °C are much lower than those obtained at *T_g_* − 20 °C, *T_g_* − 15 °C, and *T_g_* − 10 °C (Table 4, Figure 9a). Moreover, it can be noticed in Figure 9 that during loading at *T_g_* − 20 °C and *T_g_* − 15 °C, after reaching the stress maximum at the so-called “yield peak”, a drop of the stress (material softening) due to the strain localization phenomena then occurs.

As demonstrated in Figure 9a,c, during the PU-SMP unloading, the strain recovery occurs to some extent, depending on the temperature. That occurred owing to the PU-SMP structure and the responsibility of hard segments to maintain the original shape. However, a large value of residual strain (*ε_res_*) remains after the unloading at temperatures below *T_g_*, since the soft segments are frozen at low temperatures (Figure 9c).

At temperature above *T_g_*, the polymer is in the rubbery state, the soft segments are activated, and the deformed specimen recovers its original shape to a greater extent during the unloading process (Figure 9c). The reason can be attributed to the activation of the soft segments, which results in the higher entropy, internal energy, and flexibility of the polymer chains at the temperatures above *T_g_*, leading to a higher tendency of the polymer specimen to recover the deformed shape to original one [32].

The loading and unloading tangent modulus as well as the residual strain *ε_res_* mean values with standard deviations—from the mean values obtained for the PU-SMP specimens deformed at temperatures below *T_g_*, around *T_g_* and above *T_g_*—are provided in Table 4. 

#### 3.2.3. Investigation of Shape Fixity and Shape Recovery of PU-SMP in One Cycle of Thermomechanical Loading

The quantitative analysis of the shape memory properties in the PU-SMP was conducted by the thermomechanical loading program according to the procedure described in Section 2.3.2.

**Figure 10:**
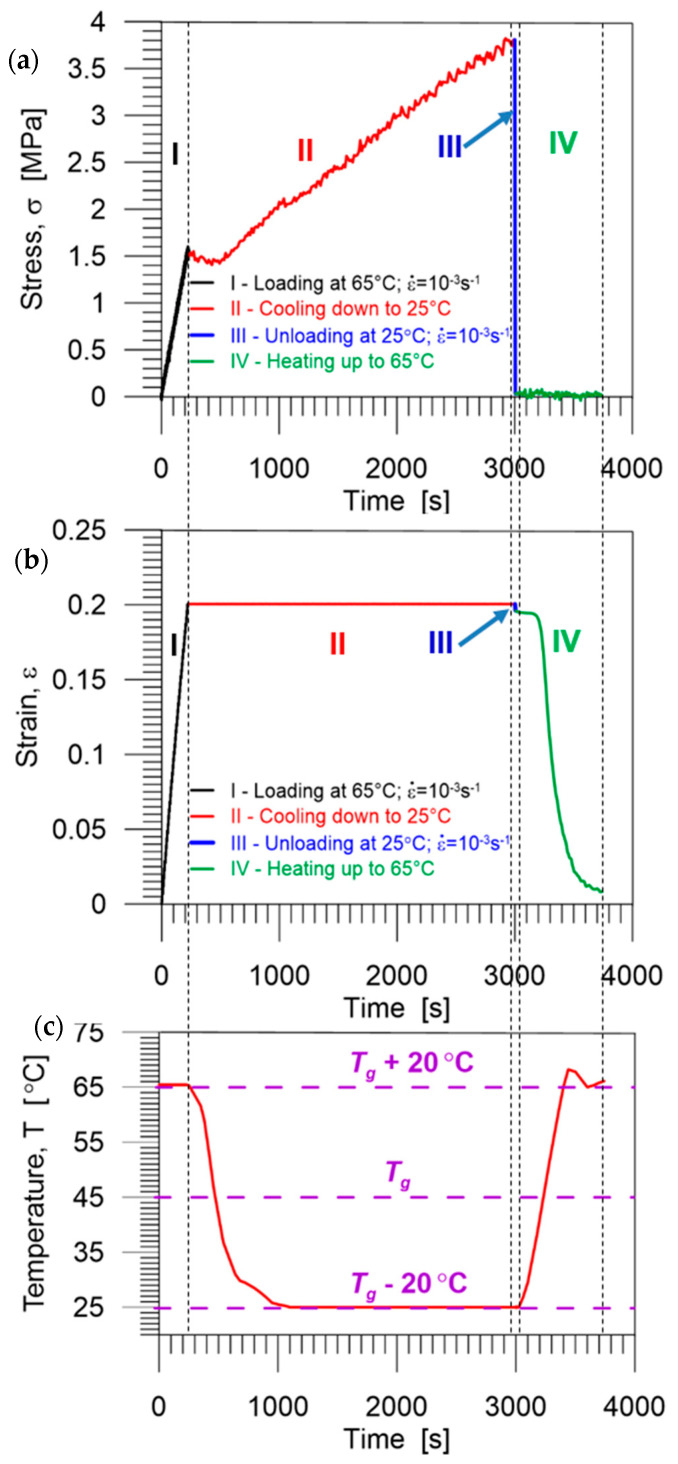
Stress (**a**), strain (**b**), and temperature (**c**) vs. time curves obtained during the subsequent stages of one thermomechanical loading cycle of the PU-SMP.

The experimental results, obtained during one cycle of thermomechanical loading program, i.e., the stress, strain, and temperature vs. time curves, are presented in Figure 10a–c. Selected colors used in the diagrams indicate each stage of the loading described in Table 1: I (black)—loading up to *ε_m_* at *T_h_* (*T_g_* + 20 °C), II (red)—cooling down to *T_l_* (*T_g_* − 20 °C), III (blue)—unloading at *T_l_*, and IV (green)—heating up to *T_h_*, respectively.

The mechanism of the shape memory behavior during the investigation can be explained by a thermodynamical approach and the PU-SMP structure evolution. By increasing the temperature above *T_g_*, the soft segments in the polymer chains between the hard segments become highly flexible and rotations of the segments around the segment bonds are significantly increased. The entropy of the system reaches a higher level, resulting in the increase of the macromolecular conformation numbers. The increase in entropy enhances the tendency of the polymer chains to turn into random coils. Applying even small loadings results in disentangling of entanglements along the polymer chains and reduces their movements, which is related to a strong decrease in the entropy (stage I). By cooling down the deformed polymer to a temperature below *T_g_* and reaching its glassy state (stage II), the movement of polymer chains is restricted and deformation is maintained after the removal of the constraints. Thus, after unloading at a temperature below *T_g_*, the fixed temporary shape is obtained (stage III). Reheating the deformed polymer to a temperature above *T_g_* releases the stress and results in turning the chains into the random coils again (stage IV). According to thermodynamic analysis, recovery of the temporally decreased entropy leads to restoration of the high entropy state. Therefore, the recovery of the previous (original) shape of the PU-SMP occurs [13,32,43].

**Figure 11:**
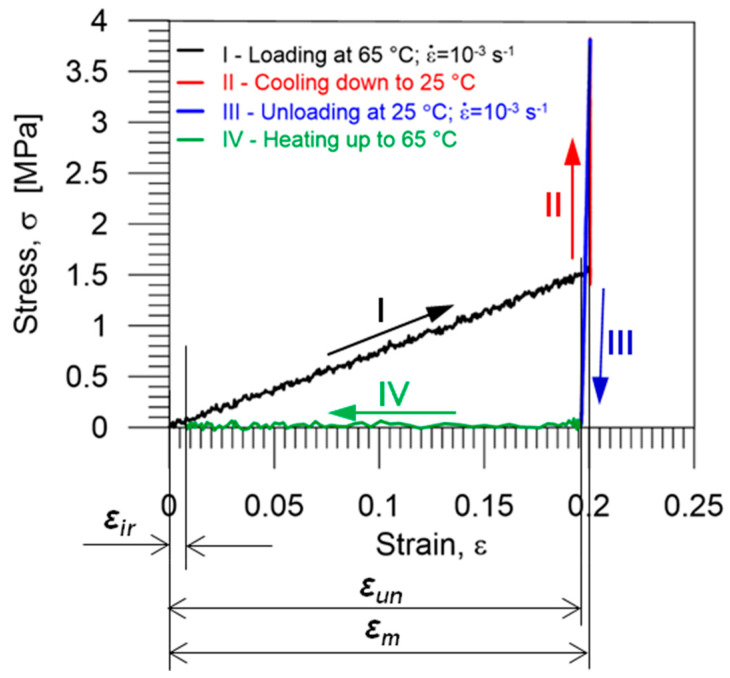
Stress vs. strain curve during the subsequent stages of one thermomechanical loading cycle; colors indicate subsequent stages I–IV of loading, as described in Table 1.

The functional parameters of shape memory polymers, crucial for their applications, namely shape fixity *R_f_* and shape recovery *R_r_*, were determined using the obtained experimental data (Figure 11) and Equations (3) and (4), proposed by H. Tobushi and S. Hayashi, e.g., [39]:

(3) $$\;R_{f}=\frac{\varepsilon_{un}}{\varepsilon_{m}}\cdot 100\%\;,$$

(4) $$R_{r}=\frac{\varepsilon_{m}-\varepsilon_{ir}}{\varepsilon_{m}}\cdot 100\%\;,$$

where

*ε_m_*—the maximum strain,
*ε_un_*—the strain obtained after unloading at *T_l_*,
*ε_ir_*—the irrecoverable strain obtained after heating up to *T_h_* under no-load conditions.

For materials with excellent shape memory properties, the values of both shape fixity and shape recovery ratios are approximately 100% [14,39,43]. However, in reality, the temporarily fixed shape resulting from cooling down the deformed specimen differs from the deformed shape obtained directly after the loading because even the chemically crosslinked network of a thermoset PU-SMP is not stable enough to fix the deformation and recover the original shape [24]. The recovered shape after heating at the temperature above *T_g_* is also slightly different from the original shape. Therefore, performing the thermomechanical loading cycle in a thermal chamber on this PU-SMP allowed us to determine the realistic values of these functional parameters. For proving the repeatability of the results, the program of the thermomechanical loading cycle was performed for five PU-SMP specimens.

The obtained shape fixity and shape recovery ratios calculated for five PU-SMP specimens are presented in Table 5 for comparison. A low discrepancy in the obtained results was found.

The average value of the shape fixity in one thermomechanical cycle is approximately 98%, which confirms the high shape fixity properties of the thermoset PU-SMP with *T_g_* = 45 °C. The mean value of the shape recovery is approximately 93%, which denotes that during the one cycle of thermomechanical loading, the shape recovery property of the PU-SMP is not as high as the shape fixity property.

#### 3.2.4. Investigation of Shape Fixity and Recovery in Three Subsequent Cycles of the PU-SMP Thermomechanical Loading Program

Determination of *R_f_* and *R_r_* of a shape memory polymer in the *N*th thermomechanical cycle is important since it allows one to predict the behavior of the shape memory material, assuring the functionality of the PU-SMP in multiple thermomechanical cycles and the repeatability of shape memory behavior. In the literature [15,16,40,41,42,43], the cyclic thermomechanical behavior has been investigated mostly in thermoplastic PU-SMP, whereas, in this paper, the shape memory parameters of a commercial thermoset PU-SMP are determined in three subsequent thermomechanical cycles. During the experiment, the applied strain rate was 10^−3^ s^−1^, while the heating and cooling rates were 4 °C/min and 7 °C/min, respectively. 

**Figure 12:**
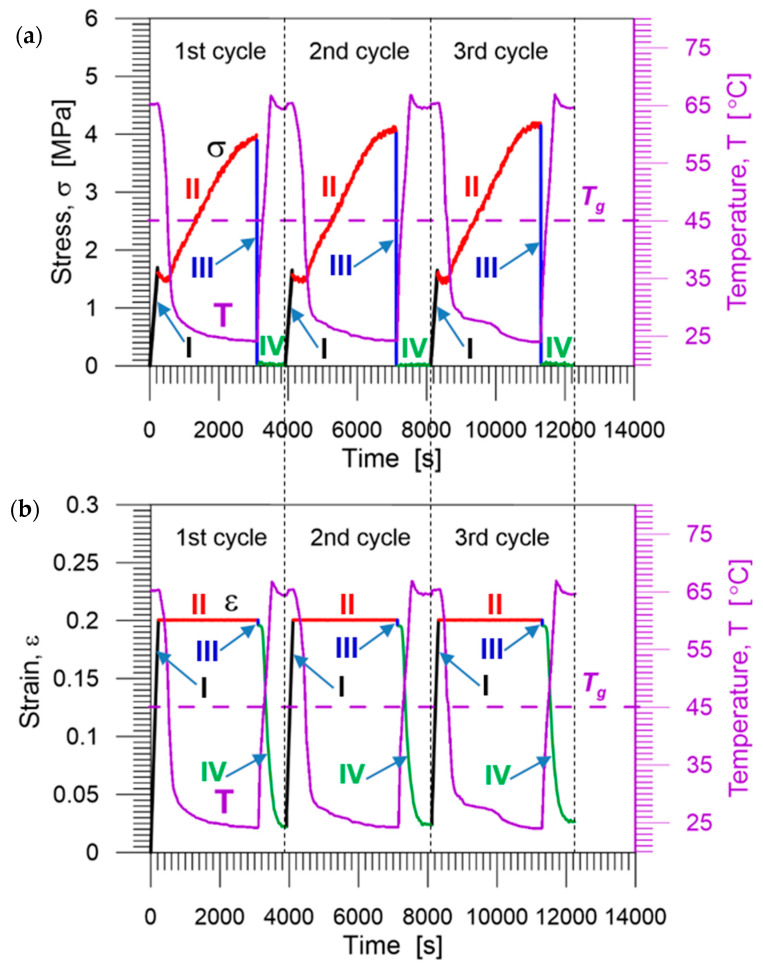
Experimental results obtained during three subsequent thermomechanical loading cycles of the PU-SMP: (**a**) stress and temperature vs. time; and (**b**) strain and temperature vs. time. Colors indicate each stage of the loading: I (black)—loading up to *ε_m_* at temperature *T_h_* (*T_g_* + 20 °C), II (red)—cooling down to *T_l_* (*T_g_* − 20 °C), III (blue)—unloading at *T_l_*, and IV (green)—heating up to *T_h_*.

The stress and temperature vs. time curves obtained for one of the PU-SMP specimens during three thermomechanical cycles are presented in Figure 12a, and strain and temperature vs. time curves are shown in Figure 12b. The experiment was repeated for five specimens. No significant discrepancies between the obtained results were observed, which confirms high quality of the PU-SMP used.

**Figure 13:**
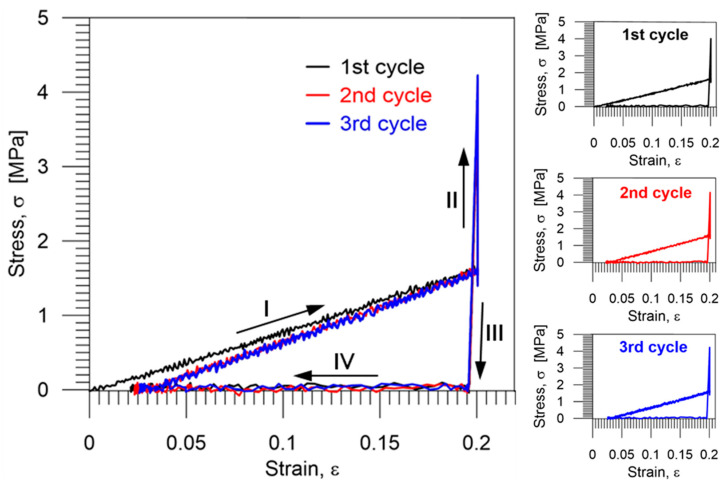
Stress vs. strain curves obtained during three subsequent thermomechanical loading cycles of the PU-SMP: I—loading up to *ε_m_* at temperature *T_h_*, II—cooling down to *T_l_*, III—unloading at *T_l_*, and IV—second heating up to *T_h_*; colors denote the cycle number.

The stress–strain curves obtained during three thermomechanical loading cycles of the PU-SMP specimen are depicted in Figure 13.

As can be noticed in Figure 12 and Figure 13, the PU-SMP behavior recorded during the second cycle is different from that recorded during the first one. However, in the third cycle, the obtained characteristics are quite similar to the second one. This implies that the deformation process, as well as the restoration of the original shape, becomes more repeatable after the second loading cycle.

Values of shape fixity (*R_f_*) and shape recovery (*R_r_*) ratios in the Nth cycle were calculated by using the obtained experimental data and the formulas proposed by H. Tobushi and S. Hayashi for cyclic thermomechanical loading programs in [15]:

(5) $$R_{f}\left(N\right)=\frac{\varepsilon_{un}\left(N\right)-\varepsilon_{ir}\left(N-1\right)}{\varepsilon_{m}-\varepsilon_{ir}\left(N-1\right)}\cdot 100\%;$$

(6) $$R_{r}\left(N\right)=\frac{\varepsilon_{un}\left(N\right)-\varepsilon_{ir}\left(N\right)}{\varepsilon_{un}\left(N\right)-\varepsilon_{ir}\left(N-1\right)}\cdot 100\%;$$

where

*ε_m_*—the maximum strain,
*ε_un_*—the strain obtained after unloading at *T_l_*,
*ε_ir_*—the irrecoverable strain; the strain obtained after heating up to *T_h_* under no-load,
*N*—the cycle number.

**Figure 14:**
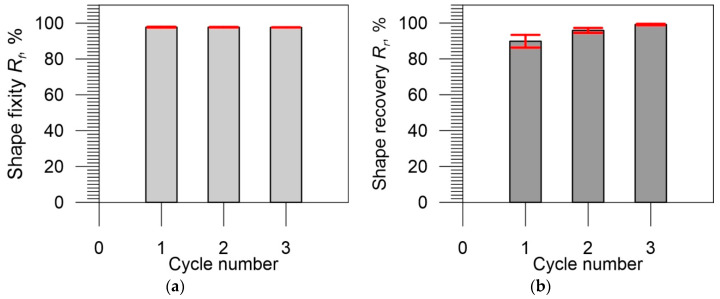
Bar diagrams presenting average values of (**a**) shape fixity and (**b**) shape recovery ratios obtained during three subsequent thermomechanical loading cycles. The error bars (in red) represent the standard deviation from the mean values of the parameters.

The obtained values of the shape fixity and shape recovery ratios in three subsequent cycles of the thermomechanical loading program for five specimens are presented in Table 6. Below the table, the average values of the shape fixity and shape recovery ratios obtained for three cycles are also presented as bar diagrams in Figure 14. The error bars represent the standard deviation of the mean values obtained from five specimens. As shown in Figure 14a, the error bars of the shape fixity ratio mean values are so small that they are negligible. Figure 14b reveals that the standard deviations of the mean values of the shape recovery ratio show a rather large discrepancy in the results in the first cycle, which decreases to lower values in the third cycle of the thermomechanical loading program.

As evidenced in Table 6 and Figure 14a, the obtained average values of the shape fixity (approximately 98%) are almost constant and are independent of the number of cycles, while the average value of the shape recovery in the first cycle is approximately 90%, increases with the number of cycles, and reaches 99% in the third cycle (Figure 14b). The stability of shape fixity and great increase of shape recovery of the thermoset PU-SMP, in comparison with thermoplastic PU-SMPs [41,42,43], can be attributed to the covalent bonds inside the network of thermoset material, which increase the thermal stability and prevent degradation of the material in thermomechanical cycles.

Kong et al. claimed that the shape recovery property of SMPs is related to strong non-covalent interactions, hard segments, and chain entanglements, whereas the shape fixity originates from the different mobility of the soft segments at the glassy and rubbery states demonstrated below and above *T_g_* [54]. When the deformed temporary shape of the PU-SMP is cooled down to the temperature below *T_g_*, the limited movements of the soft segments and presence of chemical crosslinks result in the enhancement of elastic modulus at the glassy state. A large difference in the modulus values in glassy and rubbery states, freezing of the soft segments at low temperatures, and covalent bonds in the network of the thermoset PU-SMP provide high and repeatable percentage of shape fixity of the deformed PU-SMP specimen after unloading at the temperature below *T_g_*. Therefore, the mobility of the soft segments below and above *T_g_* is independent of the number of cycles. This shows the thermal stability of the thermoset PU-SMP during the thermomechanical cycle. Accordingly, the shape fixity is mostly constant in the subsequent thermomechanical cycles.

The improvement of shape recovery by increasing the number of cycles was called the “training effect” by Tobushi et al. [15,16,39] and the “cyclic hardening” by Lendlein et al. [13]. By applying one thermomechanical cycle on PU-SMP, the reorganization of the polymer chains takes place in the direction of deformation. The decreasing of the entropy and the disentanglement of some entanglements after the thermal shape recovery in the first cycle decrease the ability of polymer chains to turn into the previous random coils [13,55]. The physical entanglements are stretched and loosened during the first cycle, and fewer entanglements remain to become disentangled after the first cycle of thermomechanical loading. Therefore, the shape recovery value during the first loading cycle is lower than those observed in the second and third cycles. The shape recovery is improved after the second and third cycles, due to fewer chain entanglements that are recovered during the second and third heating. Further research with a higher number of thermomechanical loading cycles is planned to be conducted on similar PU-SMPs.

## 4. Conclusions

A comprehensive experimental investigation conducted by dynamic mechanical analysis, differential scanning calorimetry, scanning electron microscopy, atomic force microscopy, and ultrasound testing enabled us to characterize the thermoset PU-SMP *T_g_* = 45 °C (MP4510) structure and confirm its shape memory properties.

The shape memory properties of the PU-SMP—shape fixity and shape recovery—were determined with an increased accuracy of the measurements thanks to a modified experimental setup which considered the polymer sensitivity to temperature and humidity variations, thus assuring similar conditions of the conducted investigation.

The PU-SMP shape fixity parameter was found to be approximately 98% and remained unchanged in three subsequent cycles. The shape recovery was determined to be approximately 90% in the first cycle, and it reached 99% in the third cycle due to the training effect, which can improve the shape memory properties of the PU-SMP by repeating the thermomechanical cycles.

The high value of shape fixity and its repeatability in three thermomechanical loading cycles—as well as an increase of the shape recovery obtained for the thermoset PU-SMP in comparison with thermoplastic PU-SMPs investigated in the literature—confirm high quality of the polymer and can be attributed to the covalent bonds inside the 3D network of thermoset PU-SMP, leading to its increased thermal stability.

Determination of shape fixity and shape recovery of the PU-SMP in multiple thermomechanical cycle is important since it allows one to predict the behavior of the shape memory material for assuring their effective functionality. It is especially crucial in the case of critical industrial applications, namely fast-response actuators, as well as orthopedics and post-traumatic rehabilitation devices, where high reliability, good fit, and high strength are expected.

## Figures and Tables

**Table 1:** Description of the thermomechanical loading program stages.

Preliminary Stage	I	II	III	IV
Heating up to *T_h_* = 65 °C (*T_g_* + 20 °C) (no stress), heating rate 4 °C/min	Loading up to *ε_m_* = 20% at *T_h_* = 65 °C (*T_g_* + 20 °C), strain rate $\dot{\varepsilon }$ = 10^−3^ s^−1^	Cooling down to *T_l_* = 25 °C (*T_g_* − 20 °C) (under strain *ε_m_*), cooling rate 7 °C/min, strain rate of $\dot{\varepsilon }$ = 0	Unloading to force = zero at *T_l_* = 25 °C (*T_g_* − 20 °C), strain rate of $\dot{\varepsilon }$ = 10^−3^ s^−1^	Heating up to *T_h_* = 65 °C (*T_g_* + 20 °C) (no stress), heating rate 4 °C/min

**Table 2:** DMA results of PU-SMP.

*E*′*_g_* [MPa]	*E*′*_r_* [MPa]	*E*′*_g_*/*E*′*_r_*	*T_g_* [°C]
1250	12.1	103	45

**Table 3:** Ultrasound testing results of PU-SMP.

*ρ* [g/cm^3^]	*V_L_* Mean [m/s]	*V_T_* Mean [m/s]	*E* [MPa]	*ν*
1.238	2464	953	3177 ± 35	0.412 ± 0.003

**Table 4:** Mean values of loading–unloading tangent modulus and residual strain after unloading of PU-SMP with *T_g_* = 45 °C with strain rate of 10^−2^ s^−1^ at various temperatures.

Thermal Chamber Temperature [°C]	Loading Tangent Modulus (Young’s Modulus), *E_load_* [GPa]	Unloading Tangent Modulus, *E_unload_* [GPa]	Residual Strain after Unloading, *ε_res_*
25	0.677 ± 0.015	0.481 ± 0.018	0.0541 ± 0.00005
30	0.608 ± 0.012	0.405 ± 0.011	0.0529 ± 0.00004
35	0.359 ± 0.011	0.208 ± 0.008	0.0490 ± 0.00004
45	0.0212 ± 0.0002	0.0181 ± 0.0003	0.0423 ± 0.00005
65	0.0093 ± 0.0002	0.0091 ± 0.0002	0.0195 ± 0.00004

**Table 5:** Shape fixity and shape recovery ratios calculated for five PU-SMP samples (*T_g_* = 45 °C) in one cycle of the thermomechanical loading program.

Specimen	Shape Fixity *R_f_*, %	Shape Recovery *R_r_*, %
1	98.1	87.5
2	97.9	96.0
3	97.9	94.9
4	97.8	93.8
5	97.8	92.1
Average value	97.9 ± 0.12	92.5 ± 3.32

**Table 6:** Values of shape fixity *R_f_* and shape recovery *R_r_* of the PU-SMP with *T_g_* = 45 °C in three subsequent cycles of thermomechanical loading.

Specimen	Cycle №	Shape Fixity *R_f_*, %	Shape Recovery *R_r_*, %
1	1	97.4	88.9
	2	97.5	94.9
	3	97.6	99.0
2	1	97.9	93.2
	2	97.8	95.1
	3	97.7	98.9
3	1	97.9	93.3
	2	97.8	95.1
	3	97.6	99.5
4	1	97.9	89.1
	2	97.9	96.2
	3	97.7	99.8
5	1	97.8	84.8
	2	97.7	98.2
	3	97.7	99.1
Average value	1	97.8 ± 0.22	89.9 ± 3.54
	2	97.7 ± 0.15	95.9 ± 1.38
	3	97.7 ± 0.05	99.2 ± 0.38

## Data Availability

Not applicable.
